# The Longitudinal Impact of Social Media Use on UK Adolescents' Mental Health: Longitudinal Observational Study

**DOI:** 10.2196/43213

**Published:** 2023-03-24

**Authors:** Ruth Plackett, Jessica Sheringham, Jennifer Dykxhoorn

**Affiliations:** 1 Research Department of Primary Care & Population Health University College London London United Kingdom; 2 Department of Applied Health Research University College London London United Kingdom; 3 Division of Psychiatry University College London London United Kingdom

**Keywords:** social media, mental health, depression, depressive, anxiety, adolescent, adolescence, mediation analysis, cohort study, youth, young people, self-esteem, national survey, household survey, computer use, technology use, screen time

## Abstract

**Background:**

Cross-sectional studies have found a relationship between social media use and depression and anxiety in young people. However, few longitudinal studies using representative data and mediation analysis have been conducted to understand the causal pathways of this relationship.

**Objective:**

This study aims to examine the longitudinal relationship between social media use and young people’s mental health and the role of self-esteem and social connectedness as potential mediators.

**Methods:**

The sample included 3228 participants who were 10- to 15-year-olds from Understanding Society (2009-2019), a UK longitudinal household survey. The number of hours spent on social media was measured on a 5-point scale from “none” to “7 or more hours” at the ages of 12-13 years. Self-esteem and social connectedness (number of friends and happiness with friendships) were measured at the ages of 13-14 years. Mental health problems measured by the Strengths and Difficulties Questionnaire were assessed at the ages of 14-15 years. Covariates included demographic and household variables. Unadjusted and adjusted multilevel linear regression models were used to estimate the association between social media use and mental health. We used path analysis with structural equation modeling to investigate the mediation pathways.

**Results:**

In adjusted analysis, there was a nonsignificant linear trend showing that more time spent on social media was related to poorer mental health 2 years later (n=2603, β=.21, 95% CI −0.43 to 0.84; *P*=.52). In an unadjusted path analysis, 68% of the effect of social media use on mental health was mediated by self-esteem (indirect effect, n=2569, β=.70, 95% CI 0.15-1.30; *P*=.02). This effect was attenuated in the adjusted analysis, and it was found that self-esteem was no longer a significant mediator (indirect effect, n=2316, β=.24, 95% CI −0.12 to 0.66; *P*=.22). We did not find evidence that the association between social media and mental health was mediated by social connectedness. Similar results were found in imputed data.

**Conclusions:**

There was little evidence to suggest that more time spent on social media was associated with later mental health problems in UK adolescents. This study shows the importance of longitudinal studies to examine this relationship and suggests that prevention strategies and interventions to improve mental health associated with social media use could consider the role of factors like self-esteem.

## Introduction

### Background

The World Health Organization reports that 1 in 7 adolescents (10-19 years of age) experiences mental disorders, and half of all mental illnesses begin by the age of 14 years [1,2]. The prevalence of mental disorders has increased in the last 20 years for young people, and addressing young people’s mental health has become a public health priority for many countries [3-7]. The reasons for the increase in mental disorders among young people are complex and influenced by many factors, including the use of social media. Social media can be defined as web-based networks that allow users to interact and self-present in real time or asynchronously to both broad and narrow audiences [8]. There has been rapid growth in the use of social media, from its emergence in the early 2000s to its nearly universal use 20 years later. Recent surveys show that 97% of adolescents use at least one social media platform [9]. Young people, their parents, caregivers, policymakers, clinicians, and educators have become increasingly concerned about the potential impact that increased use of social media has had on adolescent mental health [3,10,11].

Previous systematic reviews have shown an inconsistent relationship between social media use and adolescent mental health [10,12-14]. Some studies have linked increased social media use with depression, anxiety, and psychological distress, while others have found that social media can increase social support, strengthen bonds, and reduce social isolation and loneliness [10,14,15]. Many previous studies exploring the relationship between social media and mental health have been cross-sectional and may not have had representative samples [10,13,14]. Therefore, it is not well understood how the nature of the relationship between social media use and young people’s mental health changes over time, and the findings may not be generalizable. In the United Kingdom, a small number of studies using longitudinal data with representative samples have found that high levels of social media interaction were weakly associated with declines in life satisfaction, happiness, and well-being over time, particularly for female participants [16-18]. Further research is needed to understand how social media use affects adolescents’ mental health outcomes specifically and to understand who is most affected by social media and why. This could lead to the development of more tailored prevention strategies and interventions that can inform adolescents, parents and caregivers, policy makers, clinicians, and educators how to best manage the impacts of social media on mental health [19].

Previous reviews have also identified the need to explore the possible mediating factors in the relationship between social media use and mental health problems [10,20]. There is some evidence that social media use encourages young people to engage in more negative social comparison behaviors, which can lead to low self-esteem and, in turn, mental health problems [21]. Social media use can also reduce self-esteem by facilitating cyberbullying and social exclusion [22]. Therefore, self-esteem has been identified as a possible mediator of the relationship between social media use and mental health problems [10,20]. Feelings of social connectedness to peers may be another potential mediator in the link between social media and mental health problems. Social connectedness can be described as a person’s subjective experience of belonging and relatedness to others [23]. As social media platforms aim to facilitate social connections between peers, it is possible that social media use may improve feelings of peer connectedness but also lead to negative interactions, including cyberbullying, which could result in lower feelings of peer connectedness [10,22]. Few studies have explored the possible mediating role that self-esteem and peer connectedness play in explaining the relationship between social media use and mental health [24]. Exploring these mediating factors could help us understand how social media can impact mental health and inform prevention strategies and interventions to improve mental health.

### Aims

This study aims to examine the longitudinal relationship between social media use and adolescent mental health and whether this relationship is mediated by self-esteem and peer connectedness.

## Methods

### Data

This study used data from the UK Longitudinal Household Survey: Understanding Society (USoc), a nationally representative study that has interviewed all household members annually since 2009 [25]. This study used data from waves 1-10, which includes data from 2009 to 2019. The sample has been proportionately stratified and geographically clustered to identify the primary sampling units; more detail on sampling, weighting, and data collection for USoc is available [26]. This study used data from the youth self-completion questionnaire that was given to adolescents aged 10-15. Youth participation required the interviewer to ask the parent or guardian for their verbal consent and then to ask the young person for their consent [27]. A previous study found that 74% (4899) of the invited 6627 adolescents participated in the youth panel [28].

### Measures

#### Mental Health Problems

The primary outcome of the analysis was the Strengths and Difficulties Questionnaire (SDQ), a measure of children and young people’s mental health problems at the ages of 14-15 years [29]. The SDQ is a validated instrument that screens for emotional and behavioral problems in children and adolescents aged 3-16 years [30]. The SDQ is comprised of 25 items. Of these, 20 items covering hyperactivity or inattention, emotional symptoms, conduct problems, and peer relationship problems are summed to create a total difficulty score that ranges from 0 to 40. Higher scores on the total difficulties score indicate poorer mental health. SDQ total difficulties scores of 20 or above indicate a clinically-relevant risk of mental health problems [30].

#### Social Media Use

Social media use was measured by 2 questions: “Do you belong to a social website such as Bebo, Facebook, or Myspace?” and “How many hours do you spend chatting or interacting with friends through a social website like that on a normal school day?” If they responded “No” to the first question, they were coded as spending no time on social media. For all those who responded “Yes” their responses to the second question were used. Responses to the latter question were scored on a 5-point scale ranging from “none” to “7 or more hours.” Given this measure refers to active use of social media, for example, chatting and interacting with friends, this measure shall be referred to as active social media use.

#### Mediators—Self-esteem and Peer Connectedness

Self-esteem was measured using 8 questions that were measured every other wave from wave 2. The questions were: “I feel I have a number of good qualities”; “I don't have much to be proud of”; “I certainly feel useless at times”; “I am as able as most people”; “I am a likable person”; “I can usually solve my own problems”; “I am inclined to feel I am a failure”; “At times I feel I am no good at all.” These were rated on a scale from 1 to 4, with 4 being “strongly agree” and 1 being “strongly disagree.” A total mean self-esteem score was comprised of the average score across the 8 questions; some items were reverse-coded so that higher scores equated to higher self-esteem.

Peer connectedness was assessed using the following questions, which were asked at every wave: “How many close friends do you have?” and “How do you feel about your friends?” The latter question was rated on a scale from 1 to 7, with 7 being not always happy and 1 being completely happy; this was recoded so that 7 was completely happy and 1 not always happy.

#### Covariates

Covariates were chosen based on the literature and previous longitudinal analyses using the USoc data set that showed associations between these factors and social media use and mental health outcomes [16,17]. These consisted of age, sex, ethnicity of the young person, and SDQ score (mental health) at baseline. Household-level variables included the maternal highest educational attainment, the mother’s marital status, the number of people in the household who were employed, and monthly equalized household income using the Organization for Economic Cooperation and Development–modified equivalence scale. The year the adolescent took part in the survey was included as a covariate as recent analysis has shown that the prevalence of mental health problems has increased over time in the United Kingdom and because social media use has increased since the start of the survey in 2009 [4,9].

#### Analysis

We conducted unadjusted and adjusted multilevel linear regressions to explore the association between social media use and mental health. We tested for effect moderation by gender but did not find evidence for an interaction, so all estimates were not stratified by gender.

#### Mediation Analysis

**Figure 1:**
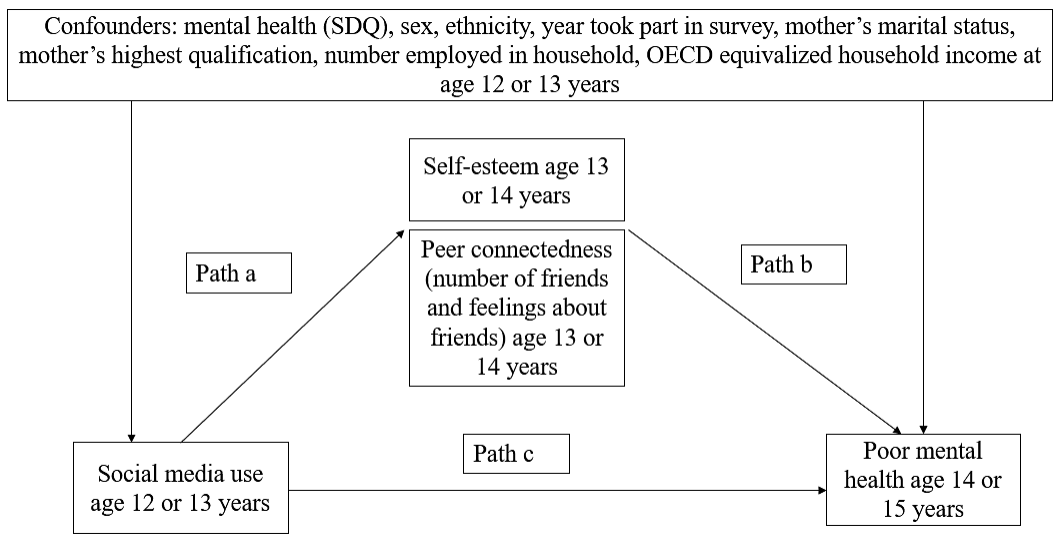
Proposed mediation model for the relationship between social media use and poor mental health. OECD: Organization for Economic Cooperation and Development; SDQ: Strengths and Difficulties Questionnaire.

We tested for mediation of the association between social media and mental health by self-esteem and peer connectedness. The proposed mediation pathway tested is shown in Figure 1. Path analysis with structural equation modeling (SEM) was used to investigate these mediation pathways and the Monte Carlo test to examine the significance of indirect effects [31]. To test for potential modification of the mediators, 3 variables were created that multiplied each of the mediators by social media use, but no moderation was found.

#### Power Calculation

Power analysis indicated that a 2-sided test with α=.05 would have 80% power to detect an odds ratio of 1.4, and the number of participants required would be 3008, based on estimates from a previous similar study [32].

#### Probability Weighting

A longitudinal enumeration weight from the last wave of the analysis provided by USoc was used to account for unequal selection probabilities, differential nonresponse, and potential sampling error. The *svy* command in Stata (StataCorp) was used to ensure the model accounted for the complex survey design.

#### Missing Data

Multiple imputations were used to account for missing data in a sensitivity analysis, where the regression analysis and SEM were conducted on imputed data after the complete case analysis. Missing data were imputed using all variables from the adjusted regression model, social media use at the ages 14-15 years, and the 3 mediator and social media use interaction variables. Several auxiliary variables were also used at baseline ages of 12-13 years and included time spent on the games console, life satisfaction across 8 domains, the number of children in the household, the mother’s age, the number of couples in the household, and the age of the first child. All analyses were conducted in Stata (version 17).

### Ethics Approval

Ethical approval was obtained from the University of Essex Ethics Committee and the Oxfordshire Research Ethics Committee (OS/HO604/124). The protocol was preregistered on Open Science Framework [33].

## Results

### Overview

Table 1 shows the baseline characteristics of the sample (n=3228) who had data for active social media use at the ages of 12-13 years and a corresponding SDQ (mental health score) at the ages of 14-15 years. Multimedia Appendix 1 presents a table describing the missingness of key variables. The complete case analysis included 40% (n=8045) of the eligible sample who participated in the survey when they were 12-13 years of age (multiple imputation sample). The sample consisted of slightly more female participants (n=1659, 51.4%) than male participants, most were of White ethnicity (n=2522, 78.1%), and most adolescents contributed their baseline scores to the USoc survey between 2009 and 2011 (n=1756, 54.4%). There were no significant differences in gender or ethnicity between those who had paired data for social media at the ages of 12-13 years and mental health scores at the ages of 14-15 years compared to those who did not.

**Table 1:** Descriptive statistics.

Baseline characteristics at ages 12-13 years (N=3228)				Participants, n (%)			Mean (SD)
	**Active social media use on weekdays**						
		None	828 (25.7)		N/A^a^		
		Less than an hour	1012 (31.4)		N/A		
		1-3 hours	1029 (31.9)		N/A		
		4-6 hours	264 (8.2)		N/A		
		7 or more hours	95 (2.9)		N/A		
	Poor mental health—SDQ^b,c^		N/A			10.45 (5.64)	
	Self-esteem^d^		N/A			3.19 (0.42)	
	Number of close friends		N/A			7.53 (7.69)	
	Happiness with friends^e^		N/A			6.37 (0.95)	
	**Gender**						
		Female	1659 (51.4)		N/A		
		Male	1569 (48.6)		N/A		
	**Ethnicity**						
		White	2522 (78.1)		N/A		
		Black African or Caribbean	146 (4.5)		N/A		
		Asian	395 (12.2)		N/A		
		Mixed	154 (4.8)		N/A		
		Other	11 (0.3)		N/A		
	**Baseline year**						
		2009-2011	1756 (54.40)		N/A		
		2012-2014	809 (25.06)		N/A		
		2015-2017	663 (20.54)		N/A		
	**Mother’s highest qualification**						
		No qualification	470 (17.8)		N/A		
		GCSE^f^ or equivalent^g^	882 (33.5)		N/A		
		A/AS^h^-level or equivalent	250 (9.5)		N/A		
		Other higher qualification	373 (14.2)		N/A		
		Degree	661 (25.1)		N/A		
	**Mother’s marital status**						
		Nonpartnered	603 (19.8)		N/A		
		Partnered	2441 (80.2)		N/A		
	**Number of employed people in household**						
		None employed	332 (10.3)		N/A		
		1 employed	1059 (32.8)		N/A		
		2 employed	1620 (50.2)		N/A		
		>2 employed	217 (6.7)		N/A		
	OECD^i^ equalized household income^j^ (£^k^)		N/A			1474.54 (971.88)	
**Mediators at ages 13-14 years (n=2642)**							
	Self-esteem		N/A			3.11 (0.44)	
	Number of close friends		N/A			7.20 (10.26)	
	How do you feel about friends?		N/A			6.22 (1.00)	
**Outcome at ages 14-15 years (n=3228)**							
	Poor mental health—SDQ		N/A			10.91 (5.81)	

^a^N/A: not applicable.

^b^Scores range from 0-40, with higher scores equating to more mental health problems.

^c^SDQ: Strengths and Difficulties Questionnaire.

^d^Scores range 1-4, higher scores equate to higher self-esteem.

^e^Scores range 1-7, higher scores equate to more happiness with friends.

^f^GCSE: General Certificate of Secondary Education.

^g^Comparison to US qualifications.

^h^A/AS level: advanced/advanced subsidiary.

^i^OECD: Organization for Economic Co-operation and Development

^j^For international income comparisons see the OECD website.

^k^US $1=0.85 GBP.

### Relationship Between Social Media and Mental Health

#### Regression Analyses

Multimedia Appendix 2 provides the pairwise correlations of all the variables. In unadjusted analyses, active social media use explained 2.4% of the variance in mental health scores, *F*_1,1858_=6.47, *P*=.01. Mental health problems, as measured on the SDQ, increased by 0.96 points for every unit increase in active social media use (95% CI 0.22-1.70), suggesting those who spend a lot of time on social media might have poorer mental health (Table 2). In the adjusted analysis that included the covariates, there was a similar trend, but this relationship was no longer significant (β=.21, *P*=.52, 95% CI −0.43 to 0.84).

In the adjusted model, the only significant predictors of mental health problems at the ages of 14-15 years were mental health problems at baseline, the year they participated in the survey, and their ethnicity. Mental health scores at the ages of 14-15 years increased by 0.53 for every unit increase in mental health scores at the ages of 12-13 years (*P*<.001, 95% CI 0.39-0.68), so poorer mental health at baseline was related to poorer mental health 2 years later. Mental health scores at 14-15 years of age increased by 0.34 for every additional year they took part in the survey (*P*<.001, 95% CI 0.17-0.50), so taking part in the survey at baseline (12-13 years) more recently was related to poorer mental health problems 2 years later. Mental health scores at ages 14-15 decreased by 3.33 for those who were Black/African Caribbean ethnicity compared to White ethnicity (*P*=.02, 95% CI, −6.09 to −0.57), so those of Black/African Caribbean ethnicity were less likely to experience mental health problems than those of White ethnicity two-years later. However, as there were few adolescents in the category of Black or African Caribbean ethnicity, there was a wide variance around this estimate.

**Table 2:** Regression analysis examining the relationship between active social media at the ages of 12-13 years and mental health at the ages of 14-15 years.

Model			Unadjusted model (N=2945)					Adjusted model (N=2603)		
			Coefficient (95% CI)		*P* value		Coefficient (95% CI)			*P* value
**Mental health—SDQ^a^ at ages 14-15 years**										
	Active social media at ages 12-13 years	0.96 (0.22 to 1.70)		.01		0.21 (−0.43 to 0.84)			.52	
	Covariates	N/A^b^		N/A		N/A			N/A	
	SDQ at ages 12-13 years	N/A		N/A		0.53 (0.39 to 0.68)			<.001	
**Gender**										
	Male	N/A		N/A		−1.12			.14	
	Female (reference)	N/A		N/A		N/A			N/A	
Year participated in survey at ages 12-13 years			N/A		N/A		0.34 (0.17 to 0.50)			<.001
**Ethnicity**										
	Black African or Caribbean	N/A		N/A		−3.33 (−6.09 to −0.57)			.02	
	Asian	N/A		N/A		−0.28 (−2.25 to 1.69)			.78	
	Mixed	N/A		N/A		−0.28 (−3.24 to 2.68)			.85	
	Other	N/A		N/A		0.61 (−7.09 to 8.31)			.87	
	White (reference)	N/A		N/A		N/A			N/A	
**Mother's highest qualification at ages 12-13 years**										
	GCSE^c^ or equivalent	N/A		N/A		0.48 (−1.7 to 2.69)			.67	
	A/AS^d^-level or equivalent	N/A		N/A		2.01 (−1.01 to 5.03)			.19	
	Other higher qualification	N/A		N/A		−1.16 (−3.38 to 1.06)			.31	
	Degree	N/A		N/A		−0.77 (−2.90 to 1.37)			.48	
	No qualifications (reference)	N/A		N/A		N/A			N/A	
**Mother's marital status at ages 12-13 years**										
	Partnered	N/A		N/A		−1.09 (−3.29 to 1.11)			.33	
	Nonpartnered (reference)	N/A		N/A		N/A			N/A	
Number of employed people in household at ages 12-13 years			N/A		N/A		−0.51 (−1.79 to 0.77)			.44
Household income at ages 12-13 years			N/A		N/A		0.00 (−0.00 to 0.00)			.57

^a^SDQ: Strengths and Difficulties Questionnaire.

^b^N/A: not applicable.

^c^GCSE: General Certificate of Secondary Education.

^d^A/AS: advanced/advanced subsidiary.

#### SEM Mediation Analyses Unadjusted Analyses

**Figure 2:**
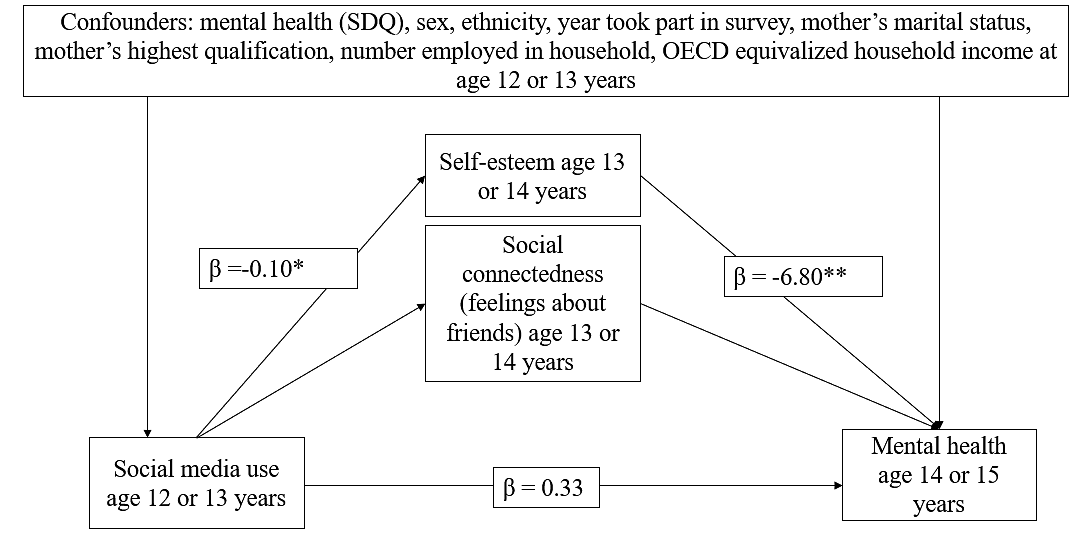
Model showing self-esteem estimates, mediating the relationship between social media use at the age of 12 or 13 years and mental health at the age of 14 or 15 years in unadjusted data. OECD: Organization for Economic Cooperation and Development; SDQ: Strengths and Difficulties Questionnaire. *P<.05, **P<.01. Coefficients unstandardized. Number of observations in unadjusted model=2569.

While there was no evidence of a longitudinal association between active social media use and mental health, we conducted a mediation analysis to test if there was mediation through self-esteem or social connectedness. The unadjusted analysis showed that more active social media use was associated with lower self-esteem (β= −.10, *P*=.01, which in turn was associated with more mental health problems (β= −6.80, *P*<.001; Table 3; Figure 2). The Monte Carlo test of the indirect effect of self-esteem on mental health problems was also significant (β=.70, *P*=.02), with 68% (0.70 over 1.03, indirect effect over total effect) of the effect of social media use being mediated by self-esteem. However, after adjusting for covariates, active social media use was no longer significantly associated with self-esteem, and the indirect effect was not significant.

In unadjusted or adjusted analyses (Table 3), the number of close friends was not associated with active social media use or poorer mental health. More active social media use was associated with a reduction in happiness with friends (β=−0.27, *P*=.01), but happiness with friends was not associated with poorer mental health (β=−0.15, *P*=.60). The Monte Carlo test of the indirect effect of happiness with friends on mental health problems was also not significant (β=.04, *P*=.64). This suggests that there was no mediation by happiness with friends and that the results did not change after adjusting for covariates.

**Table 3:** Unadjusted and adjusted structural equation modeling showing whether self-esteem and peer connectedness mediated the relationship between active social media use and mental health.

Effect				Active social media use at age 12 or 13 coefficient	Self-esteem coefficient		Number of close friends coefficient		Happiness with friends coefficient
**Unadjusted model (N=2569)**									
	**Self-esteem**								
		Direct	−0.10 (−0.18 to −0.02)^a^		N/A	N/A		N/A	
		Indirect^b^	N/A^c^		N/A	N/A		N/A	
	**Number of close friends**								
		Direct	−0.33 (−0.37 to 1.03)		2.48 (0.72 to 4.23)^d^	N/A		N/A	
		Indirect	N/A		N/A	N/A		N/A	
	**Happiness with friends**								
		Direct	−0.27 (−0.49 to −0.05)^e^		0.98 (0.47 to 1.50)^f^	N/A		N/A	
		Indirect	N/A		N/A	N/A		N/A	
	**Poor mental health at age 14 or 15 years**								
		Direct	0.33 (−0.37 to 1.03)		−6.80 (−8.22 to −5.36)^f^	−0.02 (−0.08 to 0.04)		−0.15 (−0.70 to 0.41)	
		Indirect	N/A		0.70 (0.15-1.30^g^	0.01 (−0.03 to 0.06)		0.04 (−0.12 to 0.22)	
**Adjusted model (N=2316)**									
	**Self-esteem**								
		Direct	−0.05 (−0.11 to 0.02)		N/A	N/A		N/A	
		Indirect	N/A		N/A	N/A		N/A	
	**Number of close friends**								
		Direct	−1.05 (−2.50 to −0.05)		0.68 (−1.05 to 2.41)	N/A		N/A	
		Indirect	N/A		N/A	N/A		N/A	
	**Happiness with friends**								
		Direct	−0.26 (−0.53 to 1.73)		1.11 (0.51 to 1.72)^f^	N/A		N/A	
		Indirect	N/A		N/A	N/A		N/A	
	**Poor mental health at age 14 or 15 years**								
		Direct	0.02 (−0.72 to 0.75)		−5.34 (−7.31 to −3.36)^f^	−0.05 (−0.12 to 0.02)		0.10 (−0.41 to 0.60)	
		Indirect	N/A		0.24 (−0.12 to 0.66)	0.06 (−0.03 to 0.20)		−0.03 (−0.20 to 0.12)	

^a^*P*=.01.

^b^Indirect effects are Monte Carlo estimates. Coefficients unstandardized.

^c^N/A: not applicable.

^d^*P*=.006.

^e^*P*=.01.

^f^*P*<.001.

^g^*P*=.02.

### Sensitivity Analysis

Multiple imputations used a sample of 8045 young people who participated in the survey at baseline age (12-13 years). No significant relationship was found between active social media use and mental health in the unadjusted or adjusted linear regression analyses. There was no mediation of self-esteem or peer connectedness, as there was no significant relationship between active social media use and mental health or a significant indirect effect in the unadjusted or adjusted model.

## Discussion

### Principal Findings

This study found that more time spent on active social media use at 12-13 years of age was not associated with mental health problems at 14-15 years of age for UK adolescents. Our unadjusted results suggested that more time spent on social media was associated with more mental health problems. This association was, however, fully attenuated upon adjustment for sex, ethnicity, mental health at baseline, maternal highest educational attainment, mother’s marital status, the number of people in the household who were employed, household income, and the year the adolescent took part in the survey. Much of the relationship was explained by mental health problems at baseline, the year the adolescent took part in the study, and their ethnicity. Self-esteem, but not peer connectedness, was found to be a significant mediator between social media use and mental health, but again, this relationship was attenuated after controlling for other variables and in the sensitivity analysis.

These findings are consistent with other longitudinal studies that have found modest or no evidence of a relationship between social media use and adolescent life satisfaction and well-being [16-18,34,35]. For example, a Canadian longitudinal study found that social media use did not predict depressive symptoms in female or male adolescents, but higher depressive symptoms predicted increased use of social media among girls [35]. Other studies have also explored the direction of the effect and found some evidence of a reciprocal relationship, in which poor mental health may encourage greater social media use and social media use itself may also lead to poorer mental health [17]. Cross-sectional studies have generally found negative relationships between social media use and mental health but have not been able to provide greater insight into this relationship [24,32,36]. These more methodologically robust and representative longitudinal studies are helping to build a greater understanding of the complex pathways in the relationship between social media use and mental health. Understanding these pathways could help inform recommendations on how to manage the potentially negative impacts of social media on mental health.

The complexities of measuring social media use present a major challenge to understanding the relationship between social media use and mental health in longitudinal data. Related to this complexity, social media is evolving rapidly, both in terms of the platforms used and the activities they are used for [37]. This is a challenge for longitudinal surveys, where it is desirable to ask the same question over time to ensure reliability but consequently can affect the validity of the question. For example, in the USoc, the question used to ask about social media use provides some examples of social media platforms that no longer exist (Bebo [Michael and Xochi Birch] and Myspace [eUniverse]). Adolescents are likely to be unfamiliar with these and it may affect how they answer the question. Furthermore, the social media use question in the USoc only referred to chatting and interacting with friends on social media, which is vague and difficult to interpret. The question excludes other kinds of activity, such as passive browsing and interacting with influencers or organizations. It is also unclear what is meant by terms like interaction; for example, the consequences of engaging in 1- or 2-way communication may have different impacts on mental health [38]. Future surveys should focus on measuring the behaviors that platforms allow, such as video chat, rather than specific platforms or functions, as these behaviors are less likely to change over time [37].

Previous research has also questioned whether measuring the number of hours spent on social media is a meaningful measure of social media use [34,39]. Studies are beginning to measure social media use by examining different types of it, such as passive (browsing) or active (direct messaging) use. These studies have found some evidence that different types of uses have different effects on adolescent mental health [24,34,40,41]. Passive use of social media has been associated with social comparison, envy, and feelings of anxiety and depression compared to active use [24,40,42]. In this study, there was only an active measure of social media available, which may explain why this study did not find a significant relationship between social media use and mental health outcomes. Other research has also identified 4 types of social media users: “high communicators,” “moderate communicators,” “broadcasters,” and “minimal users” [43]. Broadcasters were found to experience the poorest psychosocial outcomes compared to other user types, as they spent the most time on social media and had more passive use. The moderate use of social media was not found to have a worse impact on mental health than minimal use and moderate use may even have benefits for mental health. Overall, the evidence suggests that an optimal measure of social media use should consider both time spent on social media and the type of use [43]. Furthermore, guidance or interventions for young people with poor mental health should consider both the time and use of social media and acknowledge some of the benefits of using social media [44].

This study attempted to understand some of the complexity of the relationship between social media use and mental health by exploring the mediating roles of self-esteem and peer connectedness. Similar to previous studies, we found some evidence that poor self-esteem is an important mediator in the relationship between social media use and mental health problems [32,45]. Jiang and Ngien [45] also found that social comparison predicted poor self-esteem, which could explain why social media use may be related to poor self-esteem. Indeed, a recent model has identified that social comparison, social feedback, and self-reflection are 3 key mechanisms to explain why social media may be related to self-esteem [21]. Social media can facilitate upward social comparisons that can lead to poor self-esteem, but other research has shown that receiving social feedback on posts and engaging in self-reflective activities like curating a profile can boost self-esteem [21,46]. Other experiences on social media can also negatively impact self-esteem, such as cyberbullying and social exclusion [47]. Overall, the effects of social media on self-esteem vary by how social media is used, the nature of the content, and individual differences [19,46]. For example, several studies have suggested that females are more likely to experience poor self-esteem than males because of social media use [21,48]. Future research is needed to explore who is most affected by social media use and why, to develop more targeted prevention strategies and interventions to improve young people’s mental health. The findings of this study also indicate that interventions to reduce social media use alone may be less effective than those that also attempt to address mediating factors such as self-esteem.

This study did not find that peer connectedness was a significant mediator of the relationship between social media use and mental health problems, either by measuring this as the number of close friends or by the satisfaction with friendships. We found some indication that more active social media use was associated with feeling less happy with friendships, but happiness with friends was not associated with poorer mental health. The lack of significant findings could reflect the quality of the selected measures, which may have been inadequate to capture feelings of peer connectedness [19]. Additionally, it could be due to the paradox that social media creates for peer connectedness. On the one hand, social media use can help individuals form groups and enhance belonging, but it can also have negative outcomes, such as cyberbullying and ostracism [22,47]. Similarly, to the relationship with self-esteem, the relationship between social media and peer connectedness varies by who is using social media and how they are using it [22]. For example, a longitudinal study found that socially anxious adolescents who used social media communication to compensate for their offline social skills experienced increased loneliness, whereas those who used it to make new friends experienced reduced loneliness [49]. Further research is needed to explore the role of peer connectedness in this relationship and to explore individual differences and motivations for using social media. This could help improve guidance or recommendations for young people, parents, caregivers, clinicians, educators, and policymakers on how to manage the impacts of social media on mental health.

### Strengths and Limitations

This study has several strengths. It was based on a nationally representative sample of adolescents in the United Kingdom, so our findings are likely to be generalizable to adolescents in the United Kingdom. It was a longitudinal design that adjusted for several covariates and included a mediation analysis. This allowed for an examination of the complex relationship between social media use and mental health and associated factors. However, our study was limited by the relatively small sample size in the complete case analysis and may have been underpowered, but a sensitivity analysis was performed on a larger imputed sample where similar results were found. The analysis was also based on self-reported data and may not correlate with objective measures, such as observed time spent on social media. The measures used to assess social media use, self-esteem, and social connectedness may also have been too crude to meaningfully capture these concepts and have poor validity and reliability, but the SDQ measure of mental health has been found to be valid and reliable [30].

The study only focused on those with data at 12- to 13-year-olds and 14- to 15-year-olds, which may limit the generalizability of the findings and analysis using different ages, or time between ages and a trajectory analysis may yield different results. The age of 13 years was selected as the baseline for this study, as it is often the minimum user age for many social media platforms, but 10- to 12-year-olds also commonly report using social media, so we broadened the study to include 12- to 13-year-olds [16,50]. This also allowed us to increase the sample size in the complete case analysis as the mental health outcome was not asked at every wave. This age was also selected because it is a developmental stage in which young people’s identities are beginning to develop and peer relationships become more significant, and social networks are likely to play a key role in forming identities and relationships [41,51].

### Conclusions

This longitudinal study found little evidence to suggest that more time spent on social media is associated with later mental health problems in UK adolescents. While previous cross-sectional research suggests that there is a relationship between social media use and poor mental health, this study highlights the importance of longitudinal research in understanding these relationships, as the findings have implications for how clinicians, parents, caregivers, policymakers, and young people approach this issue. Mental health interventions or prevention strategies that address only time spent on social media may have no benefit for young people’s mental health and ignore the benefits of social media. This study also suggested that self-esteem might play a role in the relationship between social media use and mental health. Therefore, mental health interventions that focus on factors such as self-esteem and its relationship with social media could be more effective than those that solely focus on social media use. More research is needed to explore how different types of social media use affect mental health and who is most affected by social media use in order to develop more targeted prevention strategies and interventions to improve young people’s mental health.

## Appendix

Multimedia Appendix 1 Missingness by variable.

Multimedia Appendix 2 Pairwise correlations of all variables of interest.

## Abbreviations

SDQ: Strengths and Difficulties Questionnaire
SEM: structural equation modeling
USoc: Understanding Society
