# Correction: The Longitudinal Impact of Social Media Use on UK Adolescents' Mental Health: Longitudinal Observational Study

**DOI:** 10.2196/47678

**Published:** 2023-03-31

**Authors:** Ruth Plackett, Jessica Sheringham, Jennifer Dykxhoorn

**Affiliations:** 1 Research Department of Primary Care & Population Health University College London London United Kingdom; 2 Department of Applied Health Research University College London London United Kingdom; 3 Division of Psychiatry University College London London United Kingdom

In “The Longitudinal Impact of Social Media Use on UK Adolescents' Mental Health: Longitudinal Observational Study” (J Med Internet Res 2023;25:e43213), some symbols (β and −) were missing in 21 places in the paper due to an XML conversion error. The following corrections have been made:

- In the "Results" section of the Abstract, the passage “In adjusted analysis, there was a nonsignificant linear trend showing that more time spent on social media was related to poorer mental health 2 years later (n=2603, β=.21, 95% CI 0.43 to 0.84; *P*=.52). In an unadjusted path analysis, 68% of the effect of social media use on mental health was mediated by self-esteem (indirect effect, n=2569, =.70, 95% CI 0.15-1.30; *P*=.02). This effect was attenuated in the adjusted analysis, and it was found that self-esteem was no longer a significant mediator (indirect effect, n=2316, =.24, 95% CI 0.12 to 0.66; *P*=.22)” has been corrected to “In adjusted analysis, there was a nonsignificant linear trend showing that more time spent on social media was related to poorer mental health 2 years later (n=2603, β=.21, 95% CI −0.43 to 0.84; *P*=.52). In an unadjusted path analysis, 68% of the effect of social media use on mental health was mediated by self-esteem (indirect effect, n=2569, β=.70, 95% CI 0.15-1.30; *P*=.02). This effect was attenuated in the adjusted analysis, and it was found that self-esteem was no longer a significant mediator (indirect effect, n=2316, β=.24, 95% CI −0.12 to 0.66; *P*=.22).”
- In the first paragraph of the Results section subheading "Regression Analyses" within the heading "Relationship Between Social Media and Mental Health," the passage “In the adjusted analysis that included the covariates, there was a similar trend, but this relationship was no longer significant (=.21, *P*=.52, 95% CI 0.43 to 0.84)” has been changed to “In the adjusted analysis that included the covariates, there was a similar trend, but this relationship was no longer significant (β=.21, *P*=.52, 95% CI −0.43 to 0.84).”
- In the second paragraph of the Results section subheading "Regression Analyses" within the heading "Relationship Between Social Media and Mental Health," the passage “Mental health scores at the ages of 14-15 years decreased by 3.33 for those who were of Black or African Caribbean ethnicity compared to those of White ethnicity (*P*=.02, 95% CI 6.09 to 0.57), so those of Black or African Caribbean ethnicity were less likely to experience poorer mental health than those of White ethnicity 2 years later” has been changed to “Mental health scores at ages 14-15 decreased by 3.33 for those who were Black/African Caribbean ethnicity compared to White ethnicity (*P*=.02, 95% CI, −6.09 to −0.57), so those of Black/African Caribbean ethnicity were less likely to experience mental health problems than those of White ethnicity two-years later.”
- In the first paragraph of the Results section subheading "SEM Mediation Analyses Unadjusted Analyses" within the heading "Relationship Between Social Media and Mental Health," the passage “The unadjusted analysis showed that more active social media use was associated with lower self-esteem (=.10, *P*=.01, which in turn was associated with more mental health problems (=6.80, *P*<.001; Table 3; Figure 2). The Monte Carlo test of the indirect effect of self-esteem on mental health problems was also significant (=.70, *P*=.02), with 68% (0.70 or 1.03, indirect effect or total effect) of the effect of social media use being mediated by self-esteem” was changed to “The unadjusted analysis showed that more active social media use was associated with lower self-esteem (β= −.10, *P*=.01, which in turn was associated with more mental health problems (β= −6.80, *P*<.001; Table 3; Figure 2). The Monte Carlo test of the indirect effect of self-esteem on mental health problems was also significant (β=.70, *P*=.02), with 68% (0.70 over 1.03, indirect effect over total effect) of the effect of social media use being mediated by self-esteem.”
- In the second paragraph of the Results section subheading "SEM Mediation Analyses Unadjusted Analyses" within the heading "Relationship Between Social Media and Mental Health," the passage “More active social media use was associated with a reduction in happiness with friends (=.27, *P*=.01), but happiness with friends was not associated with poorer mental health (=.15, *P*=.60). The Monte Carlo test of the indirect effect of happiness with friends on mental health problems was also not significant (=.04, *P*=.64)” was changed to “More active social media use was associated with a reduction in happiness with friends (β=−0.27, *P*=.01), but happiness with friends was not associated with poorer mental health (β=−0.15, *P*=.60). The Monte Carlo test of the indirect effect of happiness with friends on mental health problems was also not significant (β=.04, *P*=.64).”

The corrections will appear in the online version of the paper on the JMIR Publications website on March 31, 2023, together with the publication of this correction notice. Because this was made after submission to PubMed, PubMed Central, and other full-text repositories, the corrected article has also been resubmitted to those repositories.

