# Supplementary material for: The Longitudinal Impact of Social Media Use on UK Adolescents' Mental Health: Longitudinal Observational Study
Source: J Med Internet Res. 2023 Mar 24;25:e43213. doi: 10.2196/43213 (PMC10132039; doi:10.2196/43213)
Supplement: Multimedia Appendix 1 [file jmir_v25i1e43213_app1.docx]

Appendix 1

Missingness by variable

| Baseline characteristics at ages 12-13 | Missing N (%) |
| --- | --- |
| Mental health - SDQ | 196 (3.0) |
| Mother’s highest qualification | 592 (18.3) |
| Mother’s marital status | 184 (5.7) |
| OECD equivalized household income | 1 (0.0) |
| Mediators at ages 13-14 |  |
| Self-esteem | 733 (22.7) |
| Number of close friends | 603 (18.7) |
| How do you feel about friends? | 586 (18.2) |
