# Supplementary material for: The Longitudinal Impact of Social Media Use on UK Adolescents' Mental Health: Longitudinal Observational Study
Source: J Med Internet Res. 2023 Mar 24;25:e43213. doi: 10.2196/43213 (PMC10132039; doi:10.2196/43213)
Supplement: Multimedia Appendix 2 [file jmir_v25i1e43213_app2.docx]

Appendix 2

Pairwise correlations of all variables of interest

| **Variables** | **(1)** | **(2)** | **(3)** | **(4)** | **(5)** | **(6)** |
| --- | --- | --- | --- | --- | --- | --- |
| (1) Social media at ages 12-13 | 1.00 |  |  |  |  |  |
| (2) SDQ at ages 14-15 | 0.15** | 1.00 |  |  |  |  |
|  | (0.00) |  |  |  |  |  |
| (3) SDQ at ages 12-13 | 0.10 ** | 0.600** | 1.00 |  |  |  |
|  | (0.00) | (0.00) |  |  |  |  |
| (4) Self-esteem at ages 13-14 | -0.09**  (0.00) | -0.48**  (0.00) | -0.44**  (0.00) | 1.00 |  |  |
|  |  |  |  |  |  |  |
| (5) Happiness with friends at ages 13-14 | - 0.00  (0.89) | -0.25**  (0.00) | -0.22**  (0.00) | 0.33**  (0.00) | 1.00 |  |
| (6) Number of close friends at ages 13-14 | 0.040*  (0.04) | -0.019  (0.34) | -0.03  (0.11) | 0.08**  (0.00) | 0.09**  (0.00) | 1.00 |

*P* values appear in parentheses below ***p*<0.001, **p*<0.05
